# Influence of Fat-Soluble Vitamin Intramuscular Supplementation on Kinematic and Morphometric Sperm Parameters of Boar Ejaculates

**DOI:** 10.3389/fvets.2022.908763

**Published:** 2022-07-12

**Authors:** Josué Calderón-Calderón, Francisco Sevilla, Eduardo R. S. Roldan, Vinicio Barquero, Anthony Valverde

**Affiliations:** ^1^Animal Reproduction Laboratory, School of Agronomy, Costa Rica Institute of Technology, Alajuela, Costa Rica; ^2^Department of Biodiversity and Evolutionary Biology, National Museum of Natural Sciences, Spanish National Research Council (CSIC), Madrid, Spain; ^3^Faculty of Agri-Food Sciences, Alfredo Volio Mata Experimental Station, University of Costa Rica, Cartago, Costa Rica

**Keywords:** spermatozoa, nutrition, fat-soluble vitamin, CASA, motility

## Abstract

Ejaculate quality can be regarded as multifactorial, with nutrition being a factor that could directly influence sperm parameters. The present study aimed to evaluate seminal quality associated with seasonal fat-soluble vitamin supplementation of boars. Seven sexually mature boars were randomly allotted to one of the three groups, and fed one of the three supplementary diets for 32 weeks: (1) control treatment (COD), without supplementation of fat-soluble vitamins, (2) treatment containing 100% fat-soluble vitamin supplementation administered intramuscularly, which was based on fat soluble vitamin supplementation (A, D3, E) (FVD1), and (3) treatment containing 50% of fat-soluble vitamin supplementation (FVD$\frac{1}{2}$). Semen was collected at 7-day intervals. Semen samples were analyzed to assess several sperm parameters using the Computer-Assisted Semen Analysis (CASA) ISAS^®^v1 system. Results showed that groups receiving FVD1 and FVD$\frac{1}{2}$ supplementation had an increased semen volume. The percentages of motile and progressively motile sperm were increased by FVD1 treatment. A statistically significant interaction between treatment and season was found in the percentage of motility and progressive motility (*p* < 0.05). Sperm concentrations showed significant differences (*p* < 0.05) between treatments. Velocity variables (VSL, VCL, and VAP) were higher (*p* < 0.05) in boars that received fat-soluble vitamin supplementation in comparison to controls receiving no supplementation. The FVD1 treatment presented spermatozoa with greater head size and more elongated heads (*p* < 0.05). Overall, the utilization of dietary fat-soluble vitamin supplementation significantly improved the semen quality of boar ejaculates. This highlights the importance of fat-soluble vitamin supplementation in sexually active boars.

## Introduction

Swine artificial insemination (AI) is performed using semen preserved in extender (1). Semen samples to be employed are subjected to several quality tests in order to maximize the production of doses for AI. Subjective semen evaluations have been replaced with objective analysis in effort to improve the assessment of fertilizing potential (2). Objective assessments provide the precision and accuracy required to obtain reliability in the estimation of quality variables (3) and would contribute to reducing technician mistakes (4). To this end, computer-assisted semen analysis (CASA) represents a valuable resource (5).

CASA technology has different modules for analyses, such as CASA-Mot (motility and kinematics) and CASA-Morph (morphometry) (6). Using CASA-Mot, spermatozoa can be classified according to their velocity as rapid, medium, slow, and static and, moreover allow for a detailed analysis of kinematic variables (7). CASA-Morph assesses morphology by using individual dimensions (length, width, area, perimeter) and shape (ellipticity, rugosity, elongation, and regularity) of the sperm head (8).

Seminal production depends on multiple factors, such as genetic improvement, reproduction techniques, health, nutrition, and handling (9). In boars, nutrition is of the utmost importance because an inadequate balance in the diet affects the libido and sperm quality (10). For this reason, supplementation must take into account season (11), age of sire (10), and weight and activity of sire (12) in order to supply the necessary mineral, vitamin, and protein requirements (13). Restrictions or deficiencies in some nutrients (14) entail a nutritional imbalance that could influence the libido and the seminal quality of the ejaculate of the reproductive boar (10).

Reactive oxygen species (ROS) are believed to be important for the normal sperm function, including processes underlying cell viability and preparation for fertilization (such as capacitation, hyperactivation, and the acrosome reaction) (15). However, sperm are susceptible to peroxidative damage due to an imbalance between ROS production and the capacity of antioxidant systems (15). The increase in ROS causes damage to the mitochondria; therefore, sperm with defective mitochondria would produce ATP inefficiently (16). In addition, an excess of ROS can also generate errors during sperm production (spermatogenesis) leading to a premature release of sperm from the germinal epithelium (17). Antioxidants can be classified into enzymatic and non-enzymatic (18). Enzymes, which are responsible for protecting sperm in the epididymis, include glutathione peroxidase (GPx), phospholipid hydroperoxide glutathione peroxidase (PHGPx), superoxide dismutase (SOD), glutathione reductase (GR) and catalase (CAT) (19). The main non-enzymatic antioxidants are vitamins A, C, and E (19).

Vitamin A is known to be necessary for the normal process of spermatogenesis (20), with retinoic acid being an alternative metabolite of vitamin A; it controls the differentiation of spermatogonia and adhesion characteristics of spermatids (14). Vitamins E and C are the most important non-enzymatic antioxidants in nutritional supplementation (21). Vitamin E includes a group of fat-soluble compounds, tocopherols, and tocotrienols, that act as antioxidants against oxidative stress (16, 22). This is because vitamin E captures free radicals, stabilizing the sperm membrane with the formation of less harmful complexes (23). High supplementation with vitamin D (2,000–4,000 Ul·Kg^−1^), is positively associated with seminal quality (24).

Seminal quality has been linked to the presence of vitamins as supplementation (24). On the other hand, deficiency of vitamins, such as vitamin E, impacts directly ATP concentration (25), sperm production, and quality of ejaculates through the swimming patterns and morphometric characteristics (26). Because of the important role of vitamins, the present study was undertaken to examine the effect of season dietary supplementation of fat-soluble vitamins on semen quality, paying particular attention to sperm morphology and kinematics.

## Materials and Methods

The use and care of animals in experimental treatments complied with the Costa Rica Institute of Technology animal welfare guidelines. Ethical approval has been given by the Committee of Centro de Investigación y Desarrollo en Agricultura Sostenible para el Trópico Húmedo at the Costa Rica Institute of Technology (CIDASTH-ITCR) according to Section 08/2020, article 1.0, DAGSC-100-2020.

### Animals and Location

The experiment was conducted at Agropecuaria Los Sagitarios S.A. commercial pig farm (Alajuela, Costa Rica) during 2020 in the Northwest of Costa Rica (Río Cuarto, 10°20′32″ N, 84°12′55″ W, Alajuela, Costa Rica). In this area, the height of the dry season is from November to April and the rainy season is from May to October. Seven sexually mature boars from a commercial terminal sire line (SL: Duroc × Pietrain) at 32.2 ± 9.8 months of age at the beginning of the experiment were used as semen donors in this study. Breeding boars were housed individually in well-ventilated pens with an average temperature of 25.8 ± 2.7°C during the time of the experiment. Data collection was performed for 32 weeks, from January 4 to August 24th 2020, with the first 2 weeks before initiating the trial allowing for adaptation to the diets.

### Diets

The animals were fed with a standard breeder mixture, containing maize, soybean meal, mineral mixture, and common salt, as ingredients to fulfill the nutrient requirements (27). Diets were mixed completely, and males were fed as a total mixed ration 2 times daily at 0,700 and 1,300 h; they consumed 2.5 kg per day, and were provided with water *ad libitum* (Table 1).

**Table 1:** Ingredients and chemical composition of diets.

**Ingredients**	**Min/Max**	**Percentage (%)**
Salt	Min.	0.4
Salt	Max.	0.5
Dry mater	Max.	88.0
Crude protein (% of DM)	Min.	16.0
Crude fat (% of DM)	Min.	2.0
Crude fiber (% of DM)	Max.	7.0
Min premix^a^		1.2
**Chemical composition**		
Ash (% of DM)	Max.	7.0
Phosphorous (% of DM)	Min.	0.7
Calcium (% of DM)	Min.	0.8
Calcium (% of DM)	Max.	1.0
Digestible energy (Mcal/kg DM)	Min.	3.3
NFE	–	56.0

*Min, minimum; Max, maximum; N.F.E, Nitrogen free extract [NFE% = 100 − (Moisture % + Crude protein % + Crude fat + Crude fiber % + Ash %)]. DM, Dry matter*.

*^a^Contained 195.0 g/kg calcium, 21.0 g/kg magnesium, 1.0 × 10^3^ mg/kg cobalt, 3.0 × 10^2^ mg/kg copper, 1.2 × 10^2^ mg/kg iodine, 3.0 × 10^3^ mg/kg iron, 2.2 × 10^3^ mg/kg manganese, 3.0 × 10^3^ mg/kg zinc, 1.1 mg/kg selenium*.

### Treatments

The animals were supplemented intramuscularly with a commercial product (Vigantol E^®^, Bayer) that provided fat-soluble vitamins. The fat-soluble vitamin supplementations were carried out monthly throughout the experiment. The experiment consisted of two treatments based on supplementation with fat-soluble vitamins (A, D3, E) and a control treatment (24). The experimental treatments included a control (COD) without fat-soluble vitamin supplementation. Treatment of FVD1 was based on the supply of 2,500,000 International Unit (IU) vitamin A, 375,000 IU vitamin D3, and 250 mg vitamin E for every 400 kg of weight (27). The treatment FVD$\frac{1}{2}$ consisted of supplementation of 50% of FVD1 (1,250,000 IU, 187,500 IU, and 125 mg of vitamins A, D3, and E, respectively, for every 400 kg of weight) (27). The assignments were completely random: two boars in the COD group, two boars in the FVD1 grouped, and three boars in the FVD$\frac{1}{2}$ group.

### Semen Collection and Evaluation

Ejaculates were collected in the morning, 1 time per week, using the “gloved-hand” technique (28) and immediately placed in a water bath at 37°C at the farm laboratory. In all cases, the sperm-rich fractions were collected and diluted with a commercial extender (Zoosperm ND5; Import-Vet, Barcelona, Spain) using the procedure described by Barquero et al. (29). Insemination doses contained a concentration of 3.7 ± 1.3 × 10^9^ spermatozoa. From each boar, 8.6 ± 4.9 ejaculates were obtained. From the treatments evaluated COD, FVD1, and FVD$\frac{1}{2}$, 11, 20, and 27 ejaculates were used, respectively. Semen samples from each ejaculate were evaluated for total motility, progressiveness, and morphology, and only ejaculates with at least 75% morphologically normal spermatozoa were used. The concentration was measured with Spermacue (Minitube, GmbH, Tiefenbach, Germany) following established protocols (30). Samples were stored at 17°C and were transported to the laboratory under the same refrigerated conditions (17°C) used for commercial distribution according to Barquero et al. (29). Semen samples (1 ml) were placed in an Eppendorf^®^ tube (Sigma-Aldrich, St. Louis, MO, USA) and remained at 37°C for 30 min before evaluations.

### Sample Preparation for Morphometric Analysis

Ejaculates from each group were assessed in duplicate for morphometric analysis. A volume of 10 μl of each sample was mixed and smeared on a glass slide and subsequently air-dried. The Diff-Quik^®^ kit (Medion Diagnostics, Düdingen, Switzerland) was used for slide staining, following the manufacturer's instructions. All slides were analyzed in a double-blind scheme.

### Assessment of Sperm Morphometry by CASA-Morph

Sperm head morphometry was analyzed using the ISAS^®^ v1 (Integrated Semen Analysis System, Proiser R + D, Valencia, Spain). The equipment consisted of a UB203 microscope (UOP/Proiser R + D) equipped with a bright-field 100× objective and a 3.3 × photo-ocular. A digital video camera (Proiser 782 m, Proiser R + D) was mounted on the microscope to capture the images and transmit them to the computer. The array size of the video frame grabber was 746 × 578 × 8 bit, providing a resolution of the analyzed images of 0.084 μm/pixel in both axes, and 256 gray levels (31). The resolution of the images was 0.08 μm per pixel in both the horizontal and vertical axes. The sperm heads were captured randomly in different fields with CASA-Morph, and only those that overlapped with background particles or other cells to interfere with the subsequent image processing were rejected as described by Barquero et al. (32). An initial erroneous definition of the sperm head boundary was corrected by varying the analysis factor of the CASA-Morph system. However, when it was not possible to obtain a correct boundary, the sperm head was deleted from the analysis.

### Assessment of Sperm Kinematics by CASA-Mot

For motility analysis, ISAS^®^D4C20 disposable counting chambers (Proiser R + D, S.L., Paterna, Spain) were used after being pre-warmed to 37°C. A volume of 2.7 μl of the diluted samples was distributed along the counting chamber fields by capillarity to fill it completely. Analyses were conducted using the CASA-Mot system ISAS^®^v1 (Integrated Semen Analysis System, Proiser R + D, Paterna, Spain) fitted with a video-camera (Proiser 782M, Proiser R + D), with 25 frames acquired per field at a frame rate of 50 Hz and final resolution of 768 × 576 pixels as described Soler (33). The camera was attached to a microscope UB203 (UOP/Proiser R + D) with a 1 × eyepiece and a 10 × negative-phase contrast objective (AN 0.25) and an integrated heated stage maintained at a constant temperature of 37.0 ± 0.5°C. The CASA settings used were a particle area between 10 and 80 μm^2^ and a connectivity of 11 μm according to Valverde (34). The percentage of total motile cells and progressive motility (%) corresponded to spermatozoa swimming forward quickly in a straight line. The following parameters defined progressive motility: straightness (STR, straightness index) ≥45% and average path velocity (VAP) ≥25 μm·s^−1^, defined as the average velocity over the smoothed cell path.

### Computerized Kinematics Analysis

The CASA-Mot variables assessed in this study included: straight-line velocity (VSL, μm·s^−1^), corresponding to the straight line from the beginning to the end of the track; curvilinear velocity (VCL, μm·s^−1^), measured over the actual point-to-point track followed by the cell; average path velocity (VAP, μm·s^−1^) the average velocity over the smoothed cell path; the amplitude of lateral head displacement (ALH, μm), defined as the maximum of the measured width of the head oscillation as the sperm swims; beat-cross frequency (BCF, Hz), defined as the frequency with which the actual track crosses the smoothed track in either direction; motility (%), defined as the percentage of total motile cells; and progressive motility (%), corresponding to spermatozoa swimming rapidly forward in a straight line as describe Soler (35). Three progression ratios, expressed as percentages, were calculated from the velocity measurements described above: linearity of forward progression (LIN = VSL/VCL·100), straightness (STR = VSL/VAP·100), and wobble (WOB = VAP/VCL·100) (36). The CASA analyses were performed in seven microscope fields on a total of at least 600 cells per sample.

### Computerized Morphometric Analysis

Images from about 200 spermatozoa from each sample were captured and analyzed, to obtain eight morphometric variable values. Following the criteria of Boersma (37), the sperm heads were measured on each slide for four primary parameters of head size [length (L, μm), width (W, μm), area (A, μm^2^), and perimeter (P, μm)] and four derived dimensionless parameters of head shape {ellipticity (L·W^−1^), rugosity [4πA·(P^2^)^−1^], elongation [(L – W)·(L + W)^−1^], and regularity [πLW·(4A)^−1^]}. Data from each individual sperm cell were saved in an Excel^®^ file (Microsoft Corporation, Redmond, Washington, USA) by the software for further analysis.

### Assessment of Morphology of Sperm Variables

A single technician carried out the assessments of sperm morphology. Sperm were classified as having normal or abnormal morphologic features following WHO strict criteria (38). A total of 200 sperm were analyzed per slide; 100 sperm from each of two different locations on the slide were assessed. If the difference between the percentage of normal sperm in the two areas was 5% or less, then the mean value was calculated (6). A subsample of each ejaculate was used to prepare one slide per sample analyzed. A total of 10 μl aliquot was placed on a glass slide and covered with a coverslip, and immediately brought to the Trumorph^®^ system (Proiser R + D, SL, Paterna, España). Trumorph^®^ exerted a constant force of 20 kiloponds (kp) uniformly distributed on the surface of the coverslip, with a temperature of 65°C. For assessment of the sample, a microscope with a 1 × eyepiece and a 40× negative-phase contrast objective was employed. Sperm morphology was examined to categorize normal cells, proximal and distal cytoplasmic droplets, or flagellum defects such as folded or coiled tails (39).

### Statistical Analysis

A normal probability plot was used to assess normal distribution. The data obtained for the analysis of all sperm variables were assessed for homoscedasticity by using the Levene test. Further, sperm variables were analyzed using the Generalized Linear Mixed Models (GLMM). The response variables were semen volume, total and progressive motility, swimming patterns (fast, average, slow, and static spermatozoa), sperm concentration, normal and abnormal sperm (%), and semen doses. A normal distribution with an identity link function was assumed for all response variables. ANOVA was further applied to evaluate statistical differences between treatments for all kinematic and morphometric variables. Other fixed factors with potential effects on sperm quality were also added to the model such as season and treatment × season interaction. A random residual effect was also added to the model to account for correlations between different ejaculates obtained from the same boar. The threshold for significance was defined as *p* < 0.05. Pairwise comparisons between season and treatment means were performed by the Tukey–Kramer test. Results were presented as mean ± standard deviation of the mean. All data were analyzed using the IBM SPSS package, version 23.0 for Windows (SPSS Inc., Chicago, IL, USA).

## Results

### Semen Characteristics

**Figure 1:**
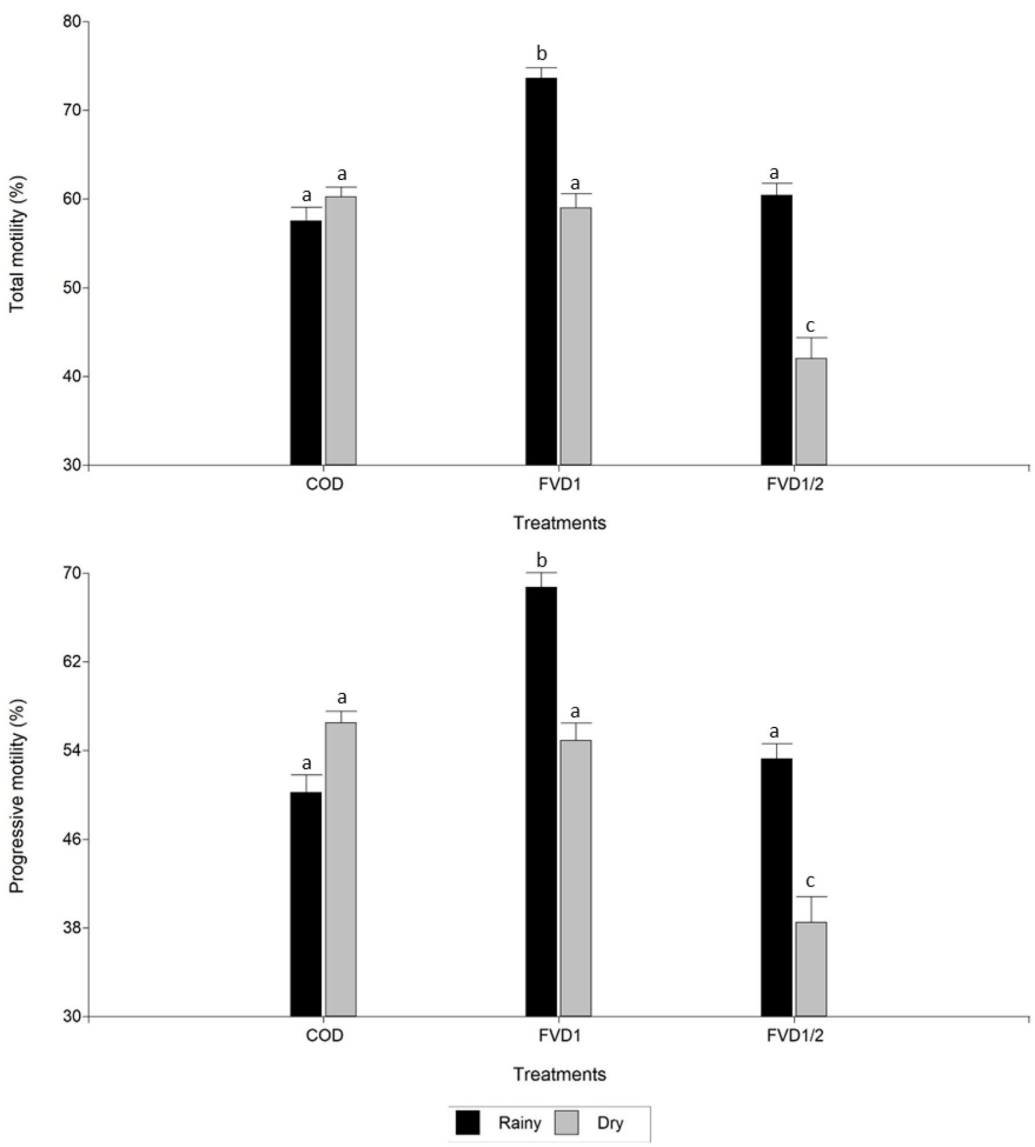
Total and progressive motilities of boar semen by season according to dietary fat-soluble vitamin supplementation. Data are expressed as the mean ± standard error of the mean. COD, control group (*n* = 11 ejaculates); FVD1, 100% fat-soluble vitamin supplementation group (*n* = 20 ejaculates); FVD$\frac{1}{2}$, 50% fat-soluble vitamin supplementation group (*n* = 27 ejaculates). ^a−c^Least square means within each treatment with differing letters differ significantly (*P* < 0.05).

**Figure 2:**
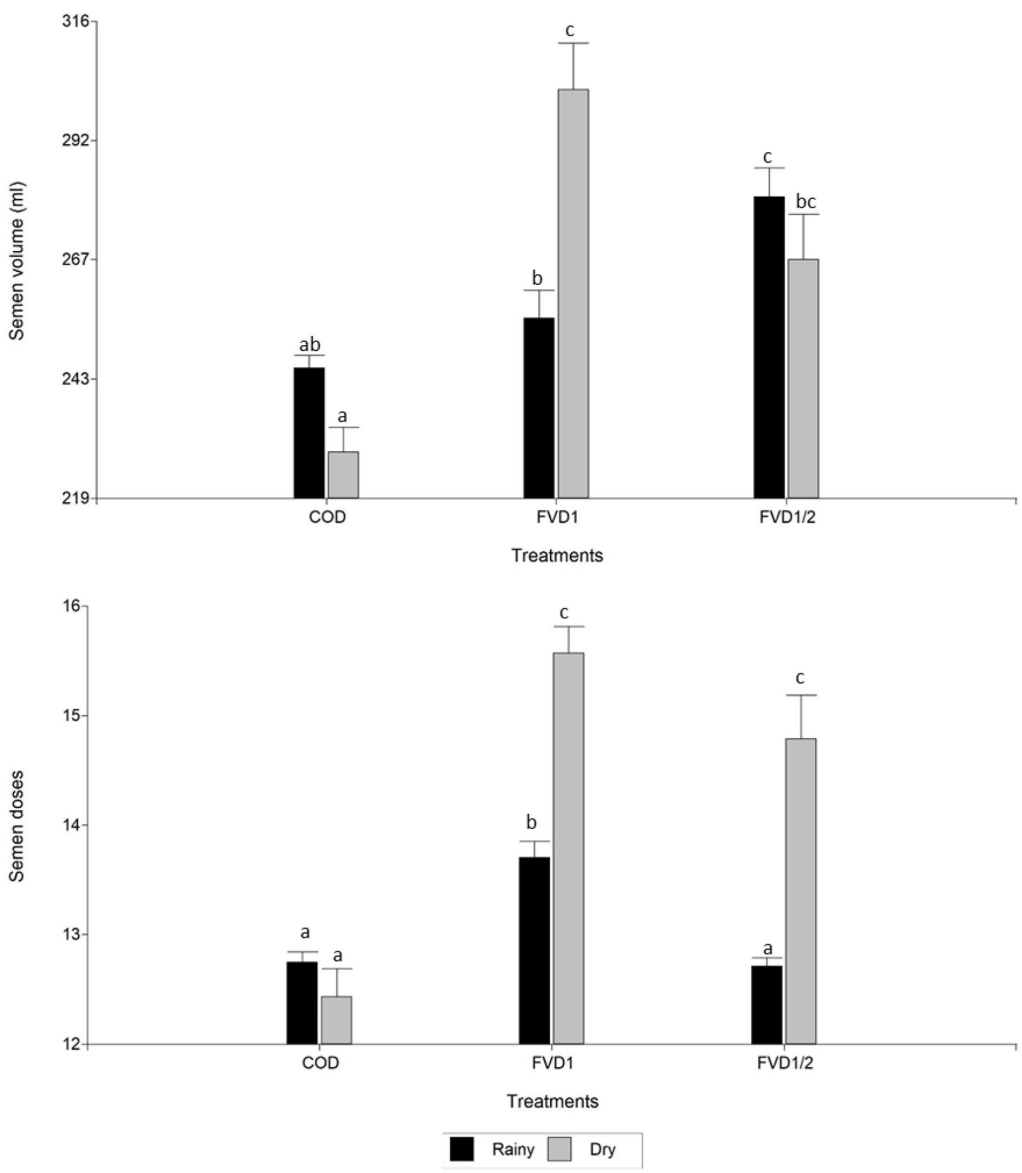
Semen volume and seminal doses produced of boar ejaculates by season according dietary fat-soluble vitamin supplementation. *n* = 58 ejaculates. Data are expressed as the mean ± standard error of the mean. COD, control group (*n* = 11 ejaculates); FVD1, 100% fat-soluble vitamin supplementation group (*n* = 20 ejaculates); FVD$\frac{1}{2}$, 50% fat-soluble vitamin supplementation group (*n* = 27 ejaculates). ^a−c^Least square means within each treatment with differing letters differ significantly (*P* < 0.05).

There was an effect of season on seminal variables analyzed (*p* < 0.05): motility, swimming patterns, morphology, and semen production doses. In the rainy season, a boar ejaculate had greater motility (total and progressive) and proportion of spermatozoa with fast movement. However, in this season a decreased number of semen doses was obtained. There were no differences (*p* > 0.05) between dry or rainy seasons for semen volume and sperm concentration (Table 2). There was an interaction between treatment × season (*p* < 0.05). In the rainy season, FVD1 treatment resulted in higher total and progressive motilities than in the dry season (Figure 1). However, it was pointed a higher semen volume and number of doses produced in the dry season with the 100% fat-soluble vitamin supplementation treatment (FVD1) (Figure 2).

**Table 2:** Overall changes in seminal characteristics (mean ± SEM) of boar ejaculates during the experiment.

**Variable**	**Season**		***P-*value**
	**Dry**	**Rainy**	
Semen volume (ml)	267.42 ± 18.76	251.20 ± 19.51	ns
Total motility (%)	50.67 ± 0.77^a^	73.77 ± 0.83^b^	**
Progressive motility (%)	45.43 ± 0.82^a^	67.63 ± 0.87^b^	**
Fast spermatozoa (%)	35.83 ± 1.34^a^	46.64 ± 0.85^b^	**
Average speed spermatozoa (%)	16.70 ± 0.49^a^	13.91 ± 0.31^b^	**
Slow speed spermatozoa (%)	2.43 ± 0.19^a^	3.53 ± 0.12^b^	**
Static spermatozoa (%)	45.04 ± 1.24^a^	35.93 ± 0.78^b^	**
Sperm concentration ( ×10^6^·ml^−1^)	267.35 ± 24.79	248.44 ± 22.85	ns
Normal sperm (%)	86.34 ± 3.29^a^	79.56 ± 2.99^b^	**
Semen doses	13.92 ± 0.48^a^	12.97 ± 0.37^b^	*
Abnormal sperm (%)	13.66 ± 3.29^a^	20.44 ± 3.00^b^	**

*n = 58 ejaculates. SEM, standard error of the mean*.

*^a, b^Least square means in a row with differing letters differ significantly (P < 0.05); ns: not significant*.

^*^P < 0.05;

^**^P < 0.001.

There was an effect (*p* < 0.05) of fat-soluble vitamin supplementation on seminal characteristics. The boars supplemented with fat-soluble vitamin presented a greater volume of ejaculate compared with the non-supplemented, control group (COD). The total and progressive motilities were higher in the FVD1 (69.93 ± 1.13%; 65.27 ± 1.15%, respectively) treatment than in FVD$\frac{1}{2}$ (56.23 ± 1.15%; 49.81 ± 1.18%, respectively) and COD (58.49 ± 1.15%; 52.60 ± 1.14%, respectively) treatments. The swimming variables indicated a higher proportion of fast spermatozoon in FVD1 in comparison to FVD$\frac{1}{2}$ and, in turn, in comparison to the COD group. There were differences (*p* < 0.05) between treatments on sperm concentration, with the COD group showing lower values (226.00 ± 4.02 × 10^6^ ml^−1^). With regards to sperm morphology, the percentage of normal spermatozoa was higher (*p* < 0.05) in treatment FVD1 (90.40 ± 1.38%) when compared to FVD$\frac{1}{2}$ (83.33 ± 2.06%) and COD (86.70 ± 1.19%) groups. There were no differences between FVD$\frac{1}{2}$ and COD treatments (*p* > 0.05) in the proportion of sperm with normal and abnormal morphology. There was a 9.3% increase in seminal doses produced in the FVD1 group in relation to the COD group. The most common morphological abnormality was distal cytoplasmatic droplets and this abnormality was lower (*p* < 0.05) in boars supplemented with FVD1 (Table 3).

**Table 3:** Effect of dietary fat-soluble vitamin supplementation on seminal characteristics (mean ± SEM) in boar ejaculates during the breeding season.

**Variable**	**Treatments** ^**1**^		
	**COD**	**FVD1**	**FVD$\frac{1}{2}$**
Semen volume (ml)	239.07 ± 4.15^a^	266.64 ± 4.31^b^	277.12 ± 4.31^b^
Total motility (%)	58.49 ± 1.15^a^	69.93 ± 1.13^b^	56.23 ± 1.15^a^
Progressive motility (%)	52.60 ± 1.14^a^	65.27 ± 1.15^b^	49.81 ± 1.18^a^
Non-progressive motility	6.04 ± 0.29^a^	4.45 ± 0.31^b^	6.27 ± 0.38^a^
Fast spermatozoa (%)	36.09 ± 1.18^a^	53.07 ± 1.23^b^	42.09 ± 1.23^c^
Average speed spermatozoa (%)	18.65 ± 0.41^a^	14.45 ± 0.43^b^	10.69 ± 0.43^c^
Slow speed spermatozoa (%)	3.81 ± 0.18^a^	2.58 ± 0.18^b^	3.22 ± 0.18^a^
Static spermatozoa (%)	41.44 ± 1.10^a^	29.90 ± 1.14^b^	44.01 ± 1.14^a^
Sperm concentration ( ×10^6^·ml^−1^)	226.00 ± 4.02^a^	240.52 ± 4.18^b^	259.64 ± 4.18^c^
Normal sperm (%)	86.70 ± 1.19^a^	90.40 ± 1.38^b^	83.33 ± 2.06^a^
Semen doses	12.67 ± 0.10^a^	13.97 ± 0.10^b^	13.16 ± 0.10^c^
Abnormal sperm (%)	13.30 ± 1.17^a^	9.60 ± 1.23^b^	16.67 ± 2.24^a^

*n = 58 ejaculates. ^1^COD, control group (n = 11 ejaculates); FVD1, 100% fat-soluble vitamin supplementation group (n = 20 ejaculates); FVD$\frac{1}{2}$, 50% fat-soluble vitamin supplementation group (n = 27 ejaculates). SEM, standard error of the mean*.

*^a−c^Least square means in a row with differing letters differ significantly (P < 0.05)*.

After 32 weeks, diets containing 100% fat-soluble vitamin supplementation (FVD1) increased the percentage of total and progressive sperm motility. The proportion of fast spermatozoa increased after 32 weeks (72.5 ± 1.8%) while there was a significant decrease of static spermatozoa from week 1 to week 32 (35.4 ± 2.4 and 13.8 ± 1.5%, respectively; Table 4).

**Table 4:** Effect of dietary fat-soluble vitamin supplementation on seminal characteristics in boar ejaculates during the experiment (least squares means ± SEM).

**Variable**	**Treatments** ^1^					
	**COD**		**FVD1**		**FVD** $\frac{1}{2}$	
	**Week 1**	**Week 32**	**Week 1**	**Week 32**	**Week 1**	**Week 32**
Semen volume (ml)	249.2 ± 5.6^y^	232.5 ± 8.0	320.0 ± 11.3^x^	258.4 ± 7.1	250.0 ± 16.0^y^	223.3 ± 9.2
Total motility (%)	57.3 ± 2.4^y^	61.0 ± 1.2^b^	64.6 ± 2.4^xy^	86.2 ± 1.5^*a^	74.1 ± 3.4^x^	76.5 ± 2.0^a^
Progressive motility (%)	54.0 ± 2.5^y^	57.1 ± 1.2^b^	61.1 ± 2.5^xy^	80.5 ± 1.6^a*^	71.0 ± 3.5^x^	69.3 ± 2.0^a^
Non-progressive motility	3.9 ± 0.3	6.1 ± 0.5	3.5 ± 0.7	5.7 ± 0.4	3.1 ± 1.0	7.2 ± 0.6*
Fast spermatozoa (%)	38.4 ± 1.4^y^	36.0 ± 2.8	45.8 ± 2.8^xy^	72.5 ± 1.8*	62.3 ± 4.0^x^	64.8 ± 2.3
Average speed spermatozoa (%)	20.1 ± 0.8	20.8 ± 2.1	16.5 ± 1.5	11.8 ± 1.0	10.2 ± 2.1	9.4 ± 1.2
Slow speed spermatozoa (%)	2.6 ± 0.3	2.5 ± 0.6	2.4 ± 0.6	1.9 ± 0.4	1.7 ± 0.8	2.3 ± 0.5
Static spermatozoa (%)	39.0 ± 1.2^y^	42.7 ± 2.4^b^	35.4 ± 2.4^y^	13.8 ± 1.5^*a^	25.9 ± 3.4^x^	23.5 ± 2.0^a^
Sperm concentration ( ×10^6^·ml^−1^)	204.8 ± 5.2^y^	200.2 ± 7.3	283.0 ± 10.3^x^	235.2 ± 6.5	193.0 ± 14.6^y^	227.3 ± 8.4
Normal sperm (%)	88.1 ± 3.2	88.3 ± 4.5	91.4 ± 6.3	86.6 ± 3.9	85.6 ± 8.8	76.1 ± 5.1
Semen doses	12.5 ± 0.2^y^	13.0 ± 0.2	15.0 ± 0.3^x^	14.0 ± 0.2	12.0 ± 0.5^y^	13.0 ± 0.2
Abnormal sperm (%)	11.9 ± 3.2	11.7 ± 4.5	8.6 ± 6.3	13.4 ± 3.9	14.4 ± 8.8	23.9 ± 5.1

*n = 58 ejaculates. ^1^COD, control group (n = 11 ejaculates); FVD1, 100% fat-soluble vitamin supplementation group (n = 20 ejaculates); FVD$\frac{1}{2}$, 50% fat-soluble vitamin supplementation group (n = 27 ejaculates). SEM, standard error of the mean*.

^*^Within treatment, significantly different from week 1 (P < 0.05).

*^x, y^Within week 1, different superscript indicates differences between treatments (P < 0.05)*.

*^a, b^Within week 32, different superscript indicates differences between treatments (P < 0.05)*.

### Overall Kinematic Variables

The velocity variables (VCL, VSL, and VAP) were lower (*p* < 0.05) in the COD group in comparison to the groups that received fat-soluble vitamin supplementation. There were no differences in the pairwise comparison by the level of supplementation (FVD1 and FVD$\frac{1}{2}$) on these kinematic variables. Similarly, for linearity (LIN), boars in the COD treatment exhibited the lowest value (58.43 ± 0.13%) in relation to the FVD1 (60.86 ± 0.13%) and FVD$\frac{1}{2}$ (60.61 ± 0.18%) groups. The straightness index (STR) was higher in the COD group compared to treatments FVD1 and FVD$\frac{1}{2}$. There were differences between the supplementation treatments for the sperm oscillation (WOB), where FVD$\frac{1}{2}$ was greater than FVD1 (68.39 ± 0.14%; 67.69 ± 0.10%, respectively). For the amplitude of lateral head displacement (ALH) and the crossover frequency (BCF), the COD treatment presented lower values compared to the FVD1 and FVD$\frac{1}{2}$ supplementation treatments (Table 5).

**Table 5:** Effect of dietary fat-soluble vitamin supplementation on kinematic sperm variables (mean ± SEM) of boar ejaculates.

	**Treatments** ^1^		
**Variable**	**COD**	**FVD1**	**FVD$\frac{1}{2}$**
VCL	57.70 ± 0.18^b^	71.40 ± 0.18^a^	71.14 ± 0.25^a^
VSL	33.65 ± 0.13^b^	42.55 ± 0.13^a^	42.18 ± 0.18^a^
VAP	38.44 ± 0.12^b^	47.18 ± 0.12^a^	47.54 ± 0.17^a^
LIN	58.43 ± 0.13^b^	60.86 ± 0.13^a^	60.61 ± 0.18^a^
STR	87.77 ± 0.11^a^	85.04 ± 0.11^c^	85.75 ± 0.15^b^
WOB	67.15 ± 0.10^c^	67.69 ± 0.10^b^	68.39 ± 0.14^a^
ALH	2.12 ± 0.01^b^	2.52 ± 0.01^a^	2.53 ± 0.01^a^
BCF	8.84 ± 0.02^c^	9.35 ± 0.02^a^	8.95 ± 0.03^b^

*n = 58 ejaculates. ^1^COD, control group (n = 11 ejaculates); FVD1, 100% fat-soluble vitamin supplementation group (n = 20 ejaculates); FVD$\frac{1}{2}$, 50% fat-soluble vitamin supplementation group (n = 27 ejaculates). SEM, standard error of the mean. VCL, curvilinear velocity (μm·s^−1^); VSL, straight-line velocity (μm·s^−1^); VAP, average path velocity (μm·s^−1^); LIN, linearity of forward progression (%); STR, straightness (%); WOB, wobble (%); ALH, amplitude of lateral head displacement (μm); BCF, beat-cross frequency (Hz); SEM, standard error of the mean*.

*^a−c^Different letters indicate differences between treatments. P < 0.05*.

### Morphometric Variables

The sperm head size variables showed differences (*p* < 0.05) between all treatments. The FVD1 group had spermatozoa with greater length, area, and head perimeter, with values of 8.93 ± 0.01, 36.91 ± 0.10, and 24.73 ± 0.03 μm, respectively. The spermatozoa from boars in the COD treatment had a greater head width (4.61 ± 0.01 μm) compared to spermatozoa in the FDV1 (4.55 ± 0.01 μm) and FVD$\frac{1}{2}$ (4.51 ± 0.01 μm) treatments. With regard to sperm head shape variables, the FVD1 treatment presented more elongated cells as indicated by the ellipticity and elongation values (1.96 ± 0.01 and 0.32 ± 0.01, respectively). The control group (COD) presented higher roughness values (0.79 ± 0.01) compared to treatments FVD1 and FVD$\frac{1}{2}$. There were differences in regularity between the COD group and FVD1 (Table 6).

**Table 6:** Morphometric variables (mean ± SEM) of size and head shape of boar sperm in different treatments with fat-soluble vitamin supplementation.

	**Treatments** ^1^		
**Variable**	**COD**	**FVD1**	**FVD$\frac{1}{2}$**
Length	8.68 ± 0.01^c^	8.93 ± 0.01^a^	8.74 ± 0.01^b^
Width	4.61 ± 0.01^a^	4.55 ± 0.01^b^	4.51 ± 0.01^c^
Area	36.05 ± 0.10^b^	36.91 ± 0.10^a^	35.59 ± 0.10^c^
Perimeter	24.22 ± 0.03^b^	24.73 ± 0.03^a^	24.21 ± 0.04^b^
Ellipticity	1.88 ± 0.01^c^	1.96 ± 0.01^a^	1.94 ± 0.01^b^
Rugosity	0.79 ± 0.01^a^	0.77 ± 0.01^c^	0.77 ± 0.01^b^
Elongation	0.30 ± 0.01^c^	0.32 ± 0.01^a^	0.31 ± 0.01^b^
Regularity	0.88 ± 0.01^a^	0.87 ± 0.01^b^	0.88 ± 0.01^a^

*n = 58 ejaculates*.

*^1^COD, control group (n = 11 ejaculates); FVD1, 100% fat-soluble vitamin supplementation group (n = 20 ejaculates); FVD$\frac{1}{2}$, 50% fat-soluble vitamin supplementation group (n = 27 ejaculates). SEM, standard error of the mean. [Length (L, μm), Width (W, μm), Area (A, μm^2^), Perimeter (P, μm)], Ellipticity (L·W^−1^), Rugosity [4πA·(P^2^)^−1^], Elongation [(L–W)·(L + W)^−1^], Regularity [πLW·(4A)^−1^]*.

*^a−c^Different letters indicate differences between treatments. P < 0.05*.

## Discussion

The volume of semen in boar ejaculates of the FVD1 group was higher than those in the FVD$\frac{1}{2}$ and COD groups. Other authors reported in boars (Duroc × Pietrain), subjected to a diet similar to the control diet of the present work, a mean ejaculate volume of 245.10 ± 3.43 ml (11), which is similar to the mean value obtained in the present study for the control treatment (COD = 239.07 ± 4.15 ml). In another study, it was demonstrated that supplementation with fat-soluble vitamins improved semen production in boars (40), but supplementation with vitamin D alone did not have significant effects (24). In other species, it has been reported that supplementation with minerals and vitamins does not have any effect (*p* > 0.05) on the ejaculate volume (41). Some studies have indicated that the higher the volume of the ejaculate, the lower the sperm concentration (42). These findings are similar to those described in this work, in which we found reduced sperm concentration in the FVD1 (*p* < 0.05) group. The higher sperm concentration in the COD and FVD$\frac{1}{2}$ groups could be due to compensations in sperm production; compensation can be a useful strategy to correct suboptimal handling conditions (43). In other species, an increase in sperm concentration of the ejaculate has been observed after supplementation with 0.35 and 0.70 ppm of Zn in comparison to animals that did not receive supplementation (44).

Morphoanomalies have relevance semen quality (45), in particular those that are the result of alterations during spermatogenesis. The data obtained in this study showed that, regardless of treatment, the most common morphological abnormality in ejaculates was distal cytoplasmatic droplets. The percentage of distal cytoplasmic droplets was lower (*p* < 0.05) in boars supplemented with FVD1. This type of abnormality can arise from the very rapid passage of sperm through the epididymis (46), compromising sperm maturation (47). This could relate also to the compensation of seminal production, with adequate fat-soluble vitamin supplementation providing conditions to produce ejaculates with high-quality spermatozoa, whereas inadequate nutrition may force sires to use immature sperm. The FVD1 treatment led to a limited percentage of abnormalities since the percentage of normal spermatozoa was >90%. It has been pointed out that it is important to maintain a threshold of 90% of normal sperm in the boar ejaculate (45).

In this study, supplementation with vitamins in the FVD1 group improved the percentage of normal cells, but the other treatments (COD and FVD$\frac{1}{2}$) did not show differences (*p* > 0.05) between them. Other studies have shown that supplementation with Zn (48) and Se (49) is necessary for normal sperm development, due to their role as cofactors of many enzymes (50). In another study examining supplementation with Se and vitamin E, a decrease in abnormal sperm was obtained with the addition of 0.5 mg of Se plus 60 mg of vitamin E per kg of feed (26). This could indicate that Se supports populations of Sertoli cells, which aid in the maturation of spermatids (14, 25). Vitamin E is an antioxidant soluble in lipids of the cell membrane, which interrupts lipid peroxidation and enhances the activity of some antioxidant enzymes that control free radicals generated during the activity of some enzymes (51). On the other hand, some authors have not reported differences (*p* > 0.05) in the percentage of normal spermatozoa when comparing treatments with supplementation with inorganic Se vs. control groups without supplementation (52).

In relation to sperm head morphometry, differences were observed after supplementation in the FVD1 treatment group. The sperm from this treatment had longer heads (8.93 ± 0.01 μm), with a greater area (36.91 ± 0.10 μm^2^) and perimeter (24.73 ± 0.03 μm), but they were narrower (4.55 ± 0.01 μm). Some authors indicated that the greater the volume of ejaculate, the larger the sperm head area (42), and with higher sperm concentration, the sperm tended to be longer and with narrower heads (53). The above could differ from the data obtained in the analysis of sperm head morphometry in this study since, although the concentration between treatments showed significant differences (*p* < 0.05), in the FVD$\frac{1}{2}$ treatment this value was higher (259.64 ± 4.18 × 10^6^ ml^−1^) than in the other treatments. In addition, in the FVD1 treatment, head length (8.93 ± 0.01 μm) and width (4.55 ± 0.01 μm) were higher than in FVD$\frac{1}{2}$, which contrasts with the values described by previous authors. The shape of the head can affect sperm movement (54, 55) since those with an elongated head move better than those with a more rounded head (55). This could explain why, in the present work, the group with the highest mean value of sperm head length presented a better value of sperm motility.

In this study, the percentage of total motility improved with supplementation in treatment FVD1, although there were no differences (*p* > 0.05) with regards to treatment FVD$\frac{1}{2}$. In a studyon boars under tropical conditions, a standard diet was administered to crossbred animals (Duroc × Pietrain) and sperm values for fast, medium, and slow movement patterns were 47.34 ± 1.51%, 22.82 ± 0.77%, and 7.42 ± 0.59%, respectively (56). These results are lower than those reported in the present work for the treatment where there was 100% fat-soluble vitamin supplementation (FVD1), with a value of 53% of the proportion of fast sperm, indicating that there is a positive effect of the supplementation on the sperm motility patterns. Trace elements are cofactors of enzymes that act as antioxidants (57), which protect sperm cells from damage caused by ROS (58). The decrease in sperm motility can be due to inappropriate use of ATP (59) and/or damage to the membrane integrity induced by ROS (60). Some authors have shown that Se helps improve sperm motility as it acts as a cofactor for the antioxidant enzyme GPx (61), and antioxidants are the most important defense against oxidative stress in the cell (51). Other studies in avian species showed that fat-soluble vitamin supplementation improves semen quality and quantity, especially sperm viability and motility (62). In our work, we determine that fat-soluble vitamin supplementation improves the total and progressive motilities, swimming parameters, and semen doses and that long term omission may result in adverse effects.

Regarding kinematic variables of velocity (VCL, VSL, VAP) and progressiveness (LIN), the supplementation with fat-soluble vitamins presented differences (*p* < 0.05) in relation to the control treatment, however, there were no differences (*p* > 0.05) between the treatments that received fat-soluble vitamin supplementation (FVD1 and FVD$\frac{1}{2}$). In a study by Lin et al. (24), an increase in curvilinear and rectilinear velocity was recorded with a supplement of 2,000 IU of vitamin D per kg of feed, compared to another treatment that consisted of a supplement with 200 IU of vitamin D per kg of feed.

When comparing boars within treatment, it was possible to observe differences between boars for all treatments of the experiment, even when they presented the same breed composition. These differences between boars can be explained by considering the ejaculate of the same boar as a heterogeneous population (29), or by the different subpopulations within each ejaculate (63) that could generate significant variability in motile sperm and kinematic patterns (64). These differences could be related to factors such as age (43), genotype (65), nutrition (22), temperature (11), and health status (66). The results obtained in this work thus show that the supplementation of fat-soluble vitamins influenced the kinematic patterns of boar spermatozoa. This suggests that reproductive management of males could include fat-soluble vitamin supplementation in boars subjected to continuous rhythms of semen collection.

A comparison of ejaculates obtained at the beginning (week 1) and end (week 32) of treatments revealed some changes in sperm parameters. The percentages of total and progressive motility showed an increase after both treatments with little change in the control (COD). An effect of fat-soluble vitamin supplementation was evident after 60–75 days of treatment, which could relate to the duration of spermatogenesis and the time of epididymal transit. Variables such as semen volume, sperm concentration, or the number of seminal doses showed a decrease in the treatment groups, and this could be explained by the frequency of semen collections, which is a well-known effect (67, 68), as well as the physical exertion that males experience over time, especially because some boars may be sensitive to increases in temperature and the observation that there may be a negative effect on spermatogenesis with temperatures above 30°C (10, 69).

Overall, studies on vitamin supplementation in relation to ejaculate quality parameters have been very scarce and many of them have only addressed the study of macroscopic characteristics (volume and concentration) of semen and few have focused on parameters such as motility, kinematics, morphology, and morphometry through CASA systems interacting with nutritional aspects. It is necessary to continue work in which the effect of the interaction between nutrition and male reproduction through CASA technology is studied, emphasizing those factors that influence the quality of ejaculates in relation to fertility.

## Conclusions

A relevant effect of fat-soluble vitamin supplementation was observed on the semen quality parameters of the boar. The restriction of vitamins causes alterations in sperm cell formation processes that increase the percentage of sperm with abnormal morphology and limits the motility and, in general, the kinematic patterns of the sperm. Furthermore, by improving motility and kinematic variables with fat-soluble vitamin supplementation, the possibility of higher fertility increases because of ejaculates with more functional spermatozoa. Furthermore, fat-soluble vitamin supplementation will likely result in seminal doses with fewer spermatozoa for an equal level of success.

## Data Availability Statement

The raw data supporting the conclusions of this article will be made available by the authors, without undue reservation.

## Ethics Statement

The animal study was reviewed and approved by Costa Rica Institute of Technology.

## Author Contributions

ER and AV: conceptualization, writing—review and editing, and visualization. JC-C and AV: methodology and investigation. JC-C: software. AV, FS, and VB: validation. AV: formal analysis, resources, data curation, supervision, project administration, and funding acquisition. JC-C, ER, VB, and AV: writing—original draft preparation. All authors listed have made a substantial, direct, and intellectual contribution to the work and agreed to the published version of the manuscript.

## Funding

This work was supported by Fundación para el Fomento y Promoción de la Investigación y Transferencia de Tecnología Agropecuaria de Costa Rica (FITTACORI) and Costa Rica Institute of Technology [Vice-Chancellor's office of Research and Extension; VIE (Vicerrectoría de Investigación y Extensión); Project-VIE-5402-2151-1015]. The funders had no role in study design, data collection, and analysis, decision to publish, or preparation of the manuscript.

## Conflict of Interest

The authors declare that the research was conducted in the absence of any commercial or financial relationships that could be construed as a potential conflict of interest.

## Publisher's Note

All claims expressed in this article are solely those of the authors and do not necessarily represent those of their affiliated organizations, or those of the publisher, the editors and the reviewers. Any product that may be evaluated in this article, or claim that may be made by its manufacturer, is not guaranteed or endorsed by the publisher.
